# 2-Phenyl­imidazolium chloride monohydrate

**DOI:** 10.1107/S1600536810006136

**Published:** 2010-02-20

**Authors:** Dao-Cheng Xia, Ji-Huan Yao

**Affiliations:** a Yuncheng University, College of Chemistry, Yuncheng 044000, People’s Republic of China

## Abstract

In the title hydrated molecular salt, C_9_H_9_N_2_^+^·Cl^−^·H_2_O, the dihedral angle between the five- and six-membered rings in the cation is 18.00 (2)°. O—H⋯Cl, N—H⋯O and N—H⋯Cl hrdrogen-bonding inter­actions are present in the crystal structure.

## Related literature

For related 2-phenyl­imidazolium nitrate structures, see: Zhang *et al.* (2007 ▶); Xia *et al.* (2009 ▶). For a phosphate salt of phenyl­imadazole, see: Xia & Yao (2010 ▶) and for a silver complex, see: Han *et al.* (2010 ▶).
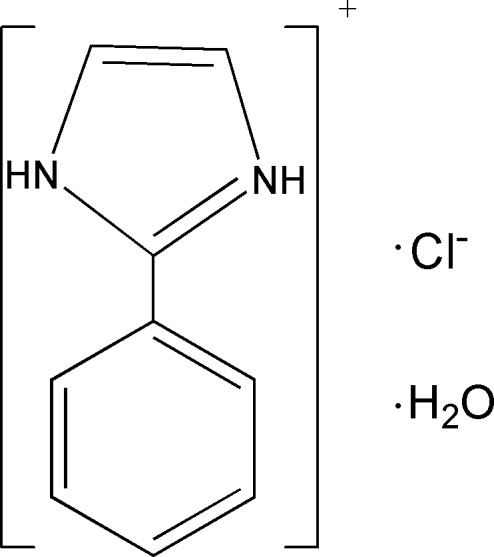


## Experimental

### 

#### Crystal data

C_9_H_9_N_2_^+^·Cl^−^·H_2_O
*M* *_r_* = 198.65
Triclinic, 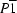

*a* = 7.2751 (10) Å
*b* = 8.8816 (13) Å
*c* = 9.3228 (10) Å
α = 105.486 (11)°
β = 106.516 (11)°
γ = 109.337 (13)°
*V* = 499.65 (15) Å^3^
*Z* = 2
Mo *K*α radiation
μ = 0.34 mm^−1^
*T* = 293 K
0.31 × 0.24 × 0.22 mm

#### Data collection

Oxford Diffraction Gemini R Ultra diffractometer
Absorption correction: multi-scan (*CrysAlis RED*; Oxford Diffraction, 2006 ▶) *T*_min_ = 0.52, *T*_max_ = 0.78
3460 measured reflections
2030 independent reflections
1198 reflections with *I* > 2σ(*I*)
*R*_int_ = 0.025

#### Refinement

*R*[*F* ^2^ > 2σ(*F* ^2^)] = 0.034
*wR*(*F* ^2^) = 0.074
*S* = 0.81
2030 reflections
126 parameters
H atoms treated by a mixture of independent and constrained refinement
Δρ_max_ = 0.16 e Å^−3^
Δρ_min_ = −0.25 e Å^−3^

### 

Data collection: *CrysAlis CCD* (Oxford Diffraction, 2006 ▶); cell refinement: *CrysAlis CCD*; data reduction: *CrysAlis RED* (Oxford Diffraction, 2006 ▶); program(s) used to solve structure: *SHELXS97* (Sheldrick, 2008 ▶); program(s) used to refine structure: *SHELXL97* (Sheldrick, 2008 ▶); molecular graphics: *SHELXTL* (Sheldrick, 2008 ▶); software used to prepare material for publication: *SHELXTL*.

## Supplementary Material

Crystal structure: contains datablocks global, I. DOI: 10.1107/S1600536810006136/om2320sup1.cif

Structure factors: contains datablocks I. DOI: 10.1107/S1600536810006136/om2320Isup2.hkl

Additional supplementary materials:  crystallographic information; 3D view; checkCIF report

## Figures and Tables

**Table 1:** Hydrogen-bond geometry (Å, °)

*D*—H⋯*A*	*D*—H	H⋯*A*	*D*⋯*A*	*D*—H⋯*A*
N1—H1⋯O1*W*	0.86	1.96	2.774 (2)	157
N2—H2⋯Cl1^i^	0.86	2.28	3.1371 (14)	172
O1*W*—H*W*11⋯Cl1	0.86 (3)	2.33 (3)	3.177 (2)	174 (2)
O1*W*—H*W*12⋯Cl1^ii^	0.88 (3)	2.32 (3)	3.190 (2)	176 (2)

Symmetry codes: (i) 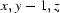; (ii) 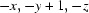.

**Fig. 1.**
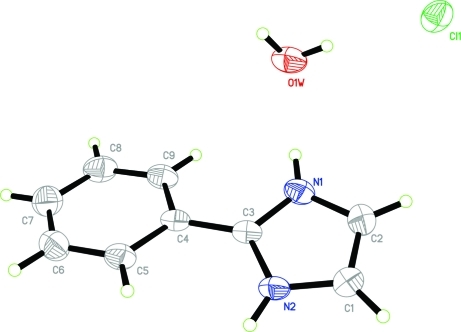
The structure of the title compound showing the atomic numbering scheme and displacement ellipsoids at the 30% probability level.

## supplementary crystallographic information

### Comment

The 2-phenylimidazolium nitrate structure has been reported as a hemihydrate (Zhang *et al.*,  2007) and as a hydrate (Xia *et al.*, 2009). Here we report the synthesis and structure of the chloride hydrate, namely, C_9_H_11_ClN_2_O.

The asymmetric unit of the title compound contains one 2-phenylimidazolium cation, one chloride anion and one water molecule (Fig. 1). There are O—H···Cl, N—H···O and N—H···Cl H-bonding interactions in the structure (Table I).

### Experimental

A mixture of 2-phenylimidazole (0.5 mmol), hydrochloric acid (0.5 mmol) and H_2_O (30 mmol) was mixed. After two weeks, colorless crystals were obtained at room temperature (22% yield).

### Refinement

All H atoms on C and N atoms were positioned geometrically (N—H = 0.86 Å and C—H = 0.93 Å) and refined as riding, with U_iso_(H)=1.2U_eq_(carrier). The water H-atom was located in a difference Fourier map, and was refined freely.

### Crystal data

**Table:**

C_9_H_9_N_2_^+^·Cl^−^·H_2_O	*Z* = 2
*M**_r_* = 198.65	*F*(000) = 208
Triclinic, *P*1	*D*_x_ = 1.320 Mg m^−^^3^
Hall symbol: -P 1	Mo *K*α radiation, λ = 0.71073 Å
*a* = 7.2751 (10) Å	Cell parameters from 2030 reflections
*b* = 8.8816 (13) Å	θ = 2.5–26.4°
*c* = 9.3228 (10) Å	µ = 0.34 mm^−^^1^
α = 105.486 (11)°	*T* = 293 K
β = 106.516 (11)°	Block, colorless
γ = 109.337 (13)°	0.31 × 0.24 × 0.22 mm
*V* = 499.65 (15) Å^3^	

### Data collection

**Table:**

Oxford Diffraction Gemini R Ultra diffractometer	2030 independent reflections
Radiation source: fine-focus sealed tube	1198 reflections with *I* > 2σ(*I*)
graphite	*R*_int_ = 0.025
Detector resolution: 10.0 pixels mm^-1^	θ_max_ = 26.4°, θ_min_ = 2.5°
ω scan	*h* = −6→9
Absorption correction: multi-scan (*CrysAlis RED*; Oxford Diffraction, 2006)	*k* = −10→11
*T*_min_ = 0.52, *T*_max_ = 0.78	*l* = −11→9
3460 measured reflections	

### Refinement

**Table:**

Refinement on *F*^2^	Primary atom site location: structure-invariant direct methods
Least-squares matrix: full	Secondary atom site location: difference Fourier map
*R*[*F*^2^ > 2σ(*F*^2^)] = 0.034	Hydrogen site location: inferred from neighbouring sites
*wR*(*F*^2^) = 0.074	H atoms treated by a mixture of independent and constrained refinement
*S* = 0.81	*w* = 1/[σ^2^(*F*_o_^2^) + (0.0394*P*)^2^] where *P* = (*F*_o_^2^ + 2*F*_c_^2^)/3
2030 reflections	(Δ/σ)_max_ < 0.001
126 parameters	Δρ_max_ = 0.16 e Å^−^^3^
0 restraints	Δρ_min_ = −0.25 e Å^−^^3^

### Special details

**Table:**

Geometry. All esds (except the esd in the dihedral angle between two l.s. planes) are estimated using the full covariance matrix. The cell esds are taken into account individually in the estimation of esds in distances, angles and torsion angles; correlations between esds in cell parameters are only used when they are defined by crystal symmetry. An approximate (isotropic) treatment of cell esds is used for estimating esds involving l.s. planes.
Refinement. Refinement of *F*^2^ against ALL reflections. The weighted *R*-factor wR and goodness of fit *S* are based on *F*^2^, conventional *R*-factors *R* are based on *F*, with *F* set to zero for negative *F*^2^. The threshold expression of *F*^2^ > σ(*F*^2^) is used only for calculating *R*-factors(gt) etc. and is not relevant to the choice of reflections for refinement. *R*-factors based on *F*^2^ are statistically about twice as large as those based on *F*, and *R*- factors based on ALL data will be even larger.

### Fractional atomic coordinates and isotropic or equivalent isotropic displacement parameters (Å^2^)

**Table:**

	*x*	*y*	*z*	*U*_iso_*/*U*_eq_	
C1	0.4640 (3)	0.2399 (2)	0.3466 (2)	0.0617 (5)	
H1A	0.5140	0.2530	0.4549	0.074*	
C2	0.4744 (3)	0.3668 (2)	0.2929 (2)	0.0653 (6)	
H2A	0.5322	0.4849	0.3570	0.078*	
C3	0.3188 (3)	0.1197 (2)	0.0774 (2)	0.0437 (4)	
C4	0.2201 (3)	−0.0061 (2)	−0.0909 (2)	0.0441 (4)	
C5	0.1124 (3)	−0.1827 (2)	−0.1299 (2)	0.0548 (5)	
H5	0.1007	−0.2215	−0.0482	0.066*	
C6	0.0231 (3)	−0.3006 (2)	−0.2889 (2)	0.0665 (6)	
H6	−0.0509	−0.4186	−0.3146	0.080*	
C7	0.0428 (3)	−0.2445 (3)	−0.4101 (3)	0.0693 (6)	
H7	−0.0167	−0.3247	−0.5174	0.083*	
C8	0.1500 (3)	−0.0708 (3)	−0.3728 (2)	0.0661 (6)	
H8	0.1634	−0.0336	−0.4551	0.079*	
C9	0.2382 (3)	0.0496 (2)	−0.2145 (2)	0.0550 (5)	
H9	0.3095	0.1676	−0.1903	0.066*	
N1	0.3844 (2)	0.29054 (16)	0.12684 (18)	0.0543 (4)	
H1	0.3719	0.3448	0.0635	0.065*	
N2	0.3658 (2)	0.08756 (17)	0.21156 (17)	0.0504 (4)	
H2	0.3385	−0.0138	0.2129	0.060*	
O1W	0.2454 (3)	0.4594 (2)	−0.0598 (3)	0.0680 (4)	
HW11	0.249 (4)	0.538 (3)	0.019 (3)	0.114 (11)*	
HW12	0.107 (5)	0.407 (3)	−0.117 (3)	0.122 (11)*	
Cl1	0.26211 (8)	0.73220 (5)	0.25005 (5)	0.0634 (2)	

### Atomic displacement parameters (Å^2^)

**Table:**

	*U*^11^	*U*^22^	*U*^33^	*U*^12^	*U*^13^	*U*^23^
C1	0.0669 (14)	0.0637 (12)	0.0518 (12)	0.0312 (10)	0.0177 (10)	0.0234 (10)
C2	0.0746 (14)	0.0527 (11)	0.0572 (14)	0.0266 (10)	0.0189 (11)	0.0164 (9)
C3	0.0437 (10)	0.0496 (10)	0.0537 (11)	0.0267 (8)	0.0245 (8)	0.0305 (8)
C4	0.0444 (10)	0.0503 (10)	0.0551 (11)	0.0291 (8)	0.0245 (9)	0.0311 (8)
C5	0.0648 (13)	0.0558 (11)	0.0580 (12)	0.0308 (10)	0.0284 (10)	0.0340 (9)
C6	0.0788 (15)	0.0552 (11)	0.0645 (14)	0.0289 (10)	0.0271 (11)	0.0255 (10)
C7	0.0787 (15)	0.0748 (14)	0.0579 (13)	0.0407 (12)	0.0277 (11)	0.0229 (10)
C8	0.0818 (15)	0.0869 (14)	0.0602 (14)	0.0500 (12)	0.0389 (11)	0.0459 (11)
C9	0.0628 (12)	0.0587 (11)	0.0663 (13)	0.0335 (10)	0.0350 (10)	0.0397 (10)
N1	0.0650 (10)	0.0474 (9)	0.0635 (11)	0.0286 (7)	0.0295 (8)	0.0324 (7)
N2	0.0566 (10)	0.0501 (8)	0.0560 (10)	0.0293 (7)	0.0218 (8)	0.0316 (7)
O1W	0.0656 (12)	0.0655 (9)	0.0919 (12)	0.0366 (8)	0.0345 (9)	0.0460 (9)
Cl1	0.0715 (3)	0.0529 (3)	0.0548 (3)	0.0174 (2)	0.0143 (2)	0.0311 (2)

### Geometric parameters (Å, °)

**Table:**

C1—C2	1.339 (2)	C6—C7	1.375 (3)
C1—N2	1.366 (2)	C6—H6	0.9300
C1—H1A	0.9300	C7—C8	1.368 (3)
C2—N1	1.362 (2)	C7—H7	0.9300
C2—H2A	0.9300	C8—C9	1.378 (3)
C3—N1	1.3282 (19)	C8—H8	0.9300
C3—N2	1.332 (2)	C9—H9	0.9300
C3—C4	1.455 (2)	N1—H1	0.8600
C4—C5	1.388 (2)	N2—H2	0.8600
C4—C9	1.392 (2)	O1W—HW11	0.86 (3)
C5—C6	1.374 (3)	O1W—HW12	0.88 (3)
C5—H5	0.9300		
			
C2—C1—N2	106.67 (17)	C7—C6—H6	119.9
C2—C1—H1A	126.7	C8—C7—C6	120.01 (19)
N2—C1—H1A	126.7	C8—C7—H7	120.0
C1—C2—N1	107.20 (16)	C6—C7—H7	120.0
C1—C2—H2A	126.4	C7—C8—C9	120.68 (17)
N1—C2—H2A	126.4	C7—C8—H8	119.7
N1—C3—N2	106.63 (14)	C9—C8—H8	119.7
N1—C3—C4	126.29 (15)	C8—C9—C4	119.67 (16)
N2—C3—C4	127.06 (15)	C8—C9—H9	120.2
C5—C4—C9	119.14 (16)	C4—C9—H9	120.2
C5—C4—C3	120.82 (15)	C3—N1—C2	109.75 (14)
C9—C4—C3	120.01 (15)	C3—N1—H1	125.1
C6—C5—C4	120.30 (16)	C2—N1—H1	125.1
C6—C5—H5	119.8	C3—N2—C1	109.74 (14)
C4—C5—H5	119.8	C3—N2—H2	125.1
C5—C6—C7	120.18 (18)	C1—N2—H2	125.1
C5—C6—H6	119.9	HW11—O1W—HW12	97 (2)

### Hydrogen-bond geometry (Å, °)

**Table:**

*D*—H···*A*	*D*—H	H···*A*	*D*···*A*	*D*—H···*A*
N1—H1···O1W	0.86	1.96	2.774 (2)	157
N2—H2···Cl1^i^	0.86	2.28	3.1371 (14)	172
O1W—HW11···Cl1	0.86 (3)	2.33 (3)	3.177 (2)	174 (2)
O1W—HW12···Cl1^ii^	0.88 (3)	2.32 (3)	3.190 (2)	176 (2)

Symmetry codes: (i) *x*, *y*−1, *z*; (ii) −*x*, −*y*+1, −*z*.
